# The nucleotide excision repair (NER) system of *Helicobacter pylori*: Role in mutation prevention and chromosomal import patterns after natural transformation

**DOI:** 10.1186/1471-2180-12-67

**Published:** 2012-05-06

**Authors:** Claudia Moccia, Juliane Krebes, Stefan Kulick, Xavier Didelot, Christian Kraft, Christelle Bahlawane, Sebastian Suerbaum

**Affiliations:** 1 Institute of Medical Microbiology and Hospital Epidemiology, Hannover Medical School, Carl-Neuberg-Str. 1, , 30625, Hannover, Germany; 2 Department of Statistics, University of Oxford, 1 South Parks Road, Oxford, OX1 3TG, UK

**Keywords:** *Helicobacter pylori*, Mutation, Recombination, Nucleotide excision repair

## Abstract

**Background:**

Extensive genetic diversity and rapid allelic diversification are characteristics of the human gastric pathogen *Helicobacter pylori*, and are believed to contribute to its ability to cause chronic infections. Both a high mutation rate and frequent imports of short fragments of exogenous DNA during mixed infections play important roles in generating this allelic diversity. In this study, we used a genetic approach to investigate the roles of nucleotide excision repair (NER) pathway components in *H. pylori* mutation and recombination.

**Results:**

Inactivation of any of the four *uvr* genes strongly increased the susceptibility of *H. pylori* to DNA damage by ultraviolet light. Inactivation of *uvrA* and *uvrB* significantly decreased mutation frequencies whereas only the *uvrA* deficient mutant exhibited a significant decrease of the recombination frequency after natural transformation. A *uvrC* mutant did not show significant changes in mutation or recombination rates; however, inactivation of *uvrC* promoted the incorporation of significantly longer fragments of donor DNA (2.2-fold increase) into the recipient chromosome. A deletion of *uvrD* induced a hyper-recombinational phenotype.

**Conclusions:**

Our data suggest that the NER system has multiple functions in the genetic diversification of *H. pylori*, by contributing to its high mutation rate, and by controlling the incorporation of imported DNA fragments after natural transformation.

## Background

The human stomach pathogen *Helicobacter pylori* infects approximately 50% of the world population, usually from childhood until old age [1]. *H. pylori* exhibits exceptionally high genetic diversity, such that almost every infected human carries one or multiple unique *H. pylori* strains [2,3]. This diversity is the result of the combination of a high mutation rate with very efficient recombination during mixed infections with multiple strains [4-7], for reviews see [8-11]. The specific mechanisms that are responsible for the high mutation rate of *H. pylori* and the unusual characteristics of its DNA uptake and recombination machinery are yet incompletely understood.

We have previously described an *in vitro* system that allows us to measure mutation and transformation frequencies in *H. pylori* wild type strains and isogenic gene knock-out mutants, as well as the length of the donor DNA fragments imported into the recipient chromosome after transformation [12]. In this system, natural transformation of different *H. pylori* wild type strains with DNA from heterologous *H. pylori* donors led to the incorporation of 1.3-3.8 kb fragments into the recipient chromosome, depending on the combination of donor and recipient strains. Imports resulting from recombination contained short interspersed sequences of the recipient (ISR) in ~10% of the cases [12,13], leading to complex mosaic patterns. The glycosylase MutY, a member of the base excision repair (BER) machinery, is involved in at least one ISR-generating pathway in *H. pylori*, repairing mismatches after the heteroduplex formation between recipient and donor DNA [12]. However, the inactivation of *mutY* in *H. pylori* did not completely abrogate the formation of ISR, suggesting that additional mechanisms might contribute to ISR generation.

In addition to BER, *H. pylori* also contains a second gap-filling DNA repair system, the nucleotide excision repair pathway (NER), whose role in *H. pylori* mutation and recombination is yet poorly understood. In *Escherichia coli*, the NER system is responsible for the replacement of bulky DNA lesions such as covalently modified bases, noncovalent drug nucleotide complexes and abasic sites generated by oxidative metabolism or ionizing radiation [14,15]. Initiation of NER starts with the recognition of DNA distortions by the UvrAB complex [16]. After recognition, UvrA dissociates and UvrC is recruited and acts as a single-stranded DNA endonuclease, cleaving at both sides of the lesion [17,18]. Finally, the unwinding activity of the UvrD helicase, which preferentially catalyzes a 3’ to 5’ unwinding, removes the excised segment. DNA polymerase I fills in the gap while the remaining nick is closed by ligase [19,20]. In *H. pylori*, orthologs of the four NER genes, *uvrA-D*, have been identified [21]; but until now, only few studies have addressed the functions of these genes. *H. pylori* UvrB was shown to be involved in the repair of acid-induced DNA damage [22], and UvrD limited homologous recombination and DNA damage-induced genomic rearrangements between DNA repeats [23].

Here we have used a genetic approach to analyze the roles of the *H. pylori* NER system components in regulating the mutation rate, and the frequency and import patterns of homologous recombination after natural transformation.

## Results

### Characterization of *H. pylori* NER mutants and their susceptibility to UV light-induced cell damage

**Figure 1:**
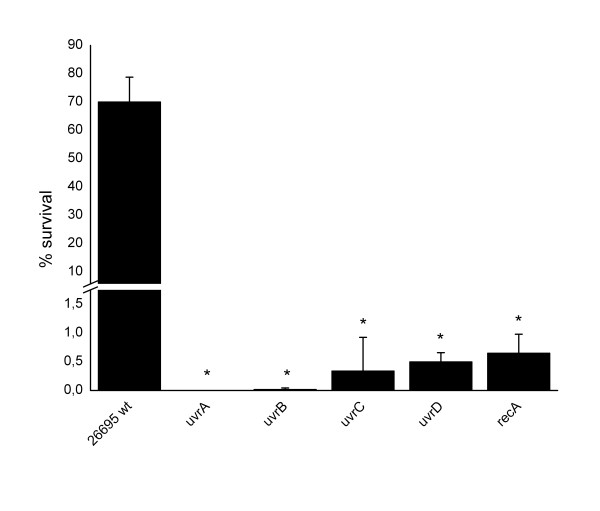
**Susceptibility of *H. pylori* NER mutants to irradiation with UV light.***H. pylori* 26695 wild type and its isogenic mutant strains were exposed to UV irradiation and the percentage of surviving cells was calculated. The data plotted represent mean ± standard deviation of at least two independent experiments. Very strongly significant results (Bayes Factor >30) are marked with an asterisk.

To investigate how the NER system contributes to genetic diversification in *H. pylori*, we individually inactivated the NER genes in *H. pylori* strain 26695 by either allelic disruption with a kanamycin resistance cassette (*uvrAuvrB*), a chloramphenicol resistance conferring cassette (*uvrC*), or by quasi-complete replacement with a kanamycin resistance (*uvrD*, see Methods for details). Since the components of the NER system participate in repairing damage caused by UV radiation in many different organisms [15], we first investigated the sensitivity of the diverse NER mutant strains against UV light. Mutants in *uvrA, uvrB, uvrC* and *uvrD* as well as a *recA* mutant [12] were exposed to UV irradiation and the amount of surviving cells was compared to the survival rate of the wt strain 26695. Inactivation of any of the NER components markedly increased the susceptibility to UV irradiation (Figure 1), indicating that all NER mutants are impaired in DNA repair.

To assess the effect of NER gene inactivation on growth properties *in vitro*, which might affect the results of other experiments reported in this study, growth curves were performed for all mutants and compared to wild type strain 26695. None of the NER mutants were affected in their growth properties in comparison with the wild type strain 26695 (Additional file 1: Figure S1).

### Spontaneous mutation frequencies in NER deficient mutants

**Figure 2:**
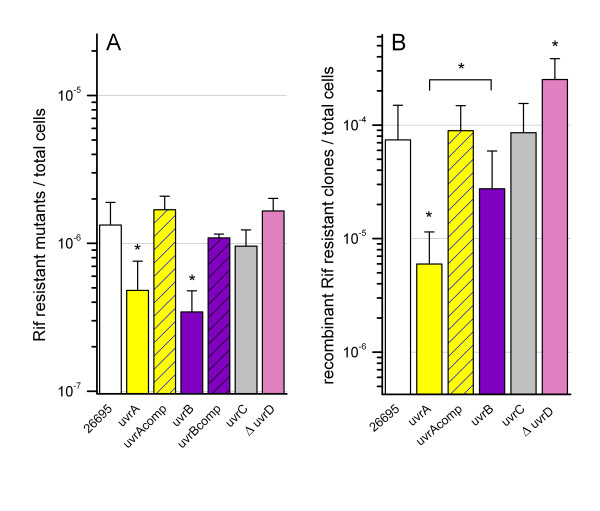
**Role of *H. pylori* NER components on mutation and recombination rates.** Frequencies of spontaneous mutations leading to Rif resistance (A) and of recombinant clones after natural transformation (B) for *H. pylori* 26695 wild type strain and isogenic NER-deficient mutants. The bars represent means ± standard deviations of three independent experiments (each experiment was performed in duplicates). Very strongly significant results (Bayes Factor >30) are marked with an asterisk.

Since the control of spontaneous mutagenesis has been associated with the NER system in *E. coli*[24], we determined the effect of inactivating the NER genes on spontaneous mutation frequencies. For this experiment, the frequencies of mutations conferring rifampicin (Rif) resistance, occurring through different single base-pair mutations in the *rpoB* gene [25], were measured (Figure 2A). The inactivation of *uvrA* and *uvrB* significantly reduced the mutation frequency, while the inactivation of *uvrC* and *uvrD* had no significant effect on the frequency of Rif resistant mutants. In order to rule out that the observed effects of the inactivation of *uvrA* and *uvrB* were due to polar effects, we constructed complemented strains where an intact copy of the target gene was introduced into the chromosome of the mutant (see Methods for details). The introduction of intact gene copies restored the mutation rates of the mutant strains to wild type levels (Figure 2A).

### Recombination frequencies in NER-deficient *H. pylori* mutants after natural transformation

We next examined the role of the *H. pylori* NER system in recombination. Each mutant strain was individually transformed with genomic DNA extracted from *H. pylori* strain J99-R3. This strain contains a point mutation (A1618T) that confers Rif resistance which can be used as a selection marker to recover recombinant clones (Additional file 2: Figure S2). Recombinant clones were distinguished from spontaneous mutants by partial *rpoB* sequence analysis. The *uvrA* mutant exhibited a highly significant decrease of the recombination frequency in comparison to the wild type (Figure 2B). A decreased mean recombination frequency was also determined for the *uvrB* deficient mutant, however, the difference between the *uvrB* mutant and wild type did not reach statistical significance (BF =14, “strong evidence”). There was no significant difference between the recombination frequency of the *uvrC* mutant and the wild type (Figure 2B). The introduction of an intact copy of the *uvrA* gene into the *uvrA* mutant restored the recombination frequency to wild type levels. In contrast, the *uvrD* deletion mutant (Δ*uvrD*) showed a hyper-recombinational phenotype (Figure 2B) that is in agreement with previous studies in *E. coli*[26] and in *H. pylori*[23].

### Characterization of the donor DNA imports after recombination in NER-deficient mutants

**Figure 3:**
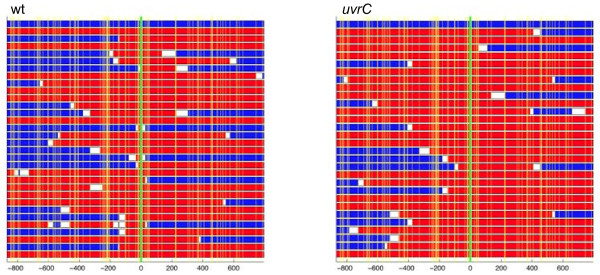
**Import patterns after transformation of recipient strain 26695 wild type (wt, left panel) and *uvrC* mutant (right panel) with DNA of Rif resistant strain J99-R3.** Each row represents a 1663 bp partial *rpoB* sequence. The blue row on top represents the sequence of the recipient strain 26695, and the red row at the bottom that of the donor strain J99-R3. Vertical yellow lines represent the positions of polymorphic sites, the green line depicts the position of the point mutation that is responsible for Rif resistance in J99-R3. Numbers below the panel: position relative to the Rif resistance point mutation, negative values indicate upstream nucleotides. The rows between 26695 and J99-R3 depict 30 sequences randomly selected from 92 clones sequenced for the wt, and all 28 *uvrC* clones analyzed for import length. Any fragment surrounded by two sites identical to the donor is shown in red, any fragment surrounded by two sites identical to the recipient is shown in blue, and the remainder of the sequence is in white. Consequently, each sequence is shown as a mosaic of colors, where blue indicates DNA from the recipient, red DNA from the donor, and white DNA of unresolved origin.

One of the characteristics of *H. pylori* is the import of relatively short fragments of donor DNA into the recipient chromosome after natural transformation. In order to understand whether components of the NER system play a role in the control of the length of DNA fragments replaced after natural transformation, and in the formation of interspersed sequences of the recipient (ISR), single recombination events were further characterized. For this, Rif resistant clones obtained using the *in vitro* transformation assay were randomly selected and a 1663 bp fragment in the *rpoB* locus was sequenced. Recombinant nucleotide sequences were aligned with both donor and recipient sequences to identify the different import parameters used for graphic comparisons of the polymorphisms (Figure 3). Maximum likelihood estimations (MLE) of the import size were calculated and the total number of ISR found among the isolates was counted. Statistical significance of the results was evaluated using a Bayesian approach (see Methods). Since the *uvrA* mutant showed a strongly reduced recombination frequency, an allele-specific PCR was used in a pre-screening step to distinguish between spontaneous mutants and recombinant clones.

There was no significant change of the import length in the *uvrA*, *uvrB*, and Δ*uvrD* mutants. Strikingly, the inactivation of *uvrC* had a strong and highly significant effect on the length of imports of donor DNA into the recipient *H. pylori* genome (Figure 3; Table 1). Indeed, the MLE of the imports increased more than 2-fold in the *uvrC* mutant compared to the wild type strain 26695 (3766 bp *vs.* 1681 bp, respectively). A functional complementation of this mutant restored this phenotype to wild type values, confirming that the generation of long imports was due to the absence of *uvrC*. None of the four mutants showed a significant change in the frequency of ISR (Table 1).

**Table 1:** **Maximum likelihood estimation (MLE) of the mean length of donor DNA imports in the *rpoB* gene and number of clones with ISR after natural transformation of *H. pylori* 26695 wild type strain and isogenic NER-deficient mutants**

		**Length of import**		**Isolates with ISR**	
**Dataset**	**Isolates**	**MLE (bp)**	**BF**	**Number**	**BF**
**26695 wt**	95	1681		9	
** *uvrA* **	26	2451	0.31	0	0.35
** *uvrB* **	24	2887	1.22	2	0.15
** *uvrC* **	28	**3766**	49.04	1	0.17
** *uvrC* comp**	35	1781	0.12	7	0.78
**Δ *uvrD* **	38	2155	0.16	6	0.33

Very strongly significant results (Bayes Factor (BF) >30) are marked in bold.

## Discussion

The nucleotide excision repair (NER) is a mechanism by which DNA lesions causing distortions of the helical structure (“bulky lesions”, induced by a variety of chemical agents and ultraviolet light) can be repaired. In *E. coli*, NER also acts on non-bulky lesions such as oxidized or methylated bases, suggesting overlapping activities of the BER and NER systems for some substrates [27,28]. The *H. pylori* genome contains orthologs of all four NER genes, *uvrA-D* (Additional file 3: Figure S3), however the function of most of these genes, and their involvement in the unusual genetic variability of this pathogen were poorly characterized. Our data show that inactivation of each of the four *H. pylori* NER genes strongly increased UV sensitivity, confirming that they are indeed functional homologs of the *E. coli* NER genes [29,30].

### Mutation rates

Inactivation of *H. pylori uvrA* and *uvrB* resulted in a significant reduction of the mutation frequency in comparison to the wild type strain. These results seem surprising, considering that one key function of the NER system is to limit mutations by repairing DNA lesions. Our results are, however, consistent with previous findings in *E. coli*, where decreased mutation frequencies were reported in *uvrA* and *uvrB* mutants after treatment with oxidized deoxyribonucleotides, while mutation rates were unaffected in a *uvrC* mutant [31]. Under non-damage-inducing conditions, *E. coli* mutants in *uvrAuvrB* and *uvrC* exhibited a lower mutation rate [24]. The excision and replacement of undamaged bases were first characterized by Branum and colleagues who showed that in *E. coli* and in human cells, NER is able to excise damage-free fragments in lengths of 12–13 and 24–32 bp, respectively [32]. This process has been referred to as “gratuitous mutations” and it has been suggested that it may be a major source of oncogene mutations in humans [15,33]. Such a double functionality of the NER proteins has been also reported for *Pseudomonas putida* and *E. coli* where the NER system is also involved in the generation of mutations [24,34]. Based on our results, we hypothesize that the basal level of NER-mediated replacement activity on undamaged DNA is contributing to the overall high mutation frequency that is characteristic of *H. pylori* and contributes to its rapid genetic diversification [4,7,10]. As outlined above, the effects of *uvrC* inactivation on mutation rates in other bacterial species are complex and depend on the experimental conditions. We note that *uvrC* does not appear to contribute to the generation of gratuitous mutations in *H. pylori*.

### The NER system has a dual role in the control of the homologous recombination in *H. pylori*

Our data show that the inactivation of *uvrA* significantly decreased the recombination frequency after natural transformation of *H. pylori*. A decrease was also observed with a *uvrB* mutant, which was suggestive (BF = 14), but did not reach statistical significance. The recombination frequency could be restored by functional complementation, indicating that UvrA facilitates homologous recombination in *H. pylori*. UvrA was not essential for this process, since recombinants were still detected in the mutant. Recombination frequencies differed significantly between *uvrA* and *uvrB* mutants, the reason of this statistically highly significant difference between both mutants remains to be elucidated.

Inactivation of UvrC likewise had no significant effect on recombination frequencies in *H. pylori.* By contrast, UvrD was found to act as an inhibitor of homologous recombination, as previously shown by other investigators [23].

We note that inactivation of *uvrC* promoted the incorporation of significantly longer DNA fragments into the *H. pylori* genome (2.2 fold increase) in comparison to the wild type strain, while a complemented mutant strain exhibited imports indistinguishable from wild type. We also observed that a different *uvrD* mutant strain, constructed by insertion of an antibiotic resistance cassette into the middle of the *uvrD* gene (and hence potentially capable of expressing a truncated UvrD protein), exhibited a strongly increased import length (data not shown). The mechanisms underlying these observations are as yet unclear.

**Figure 4:**
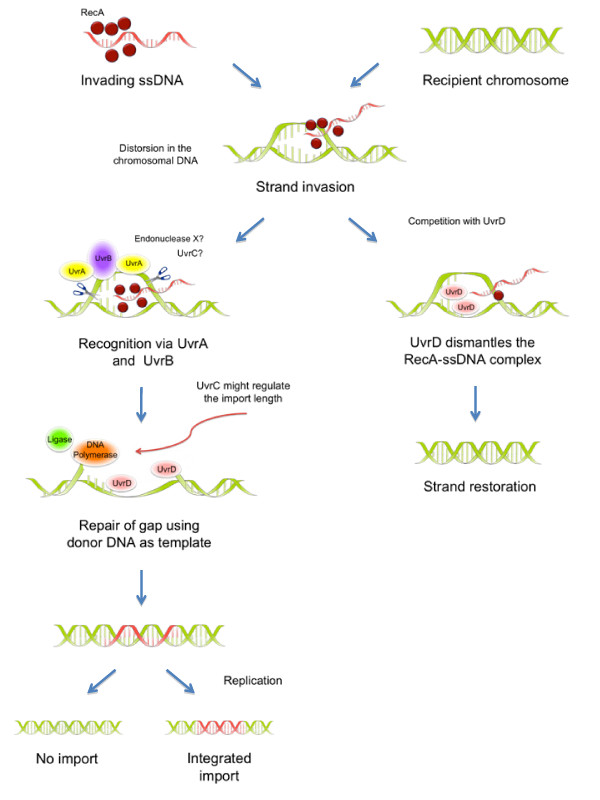
**Hypothetical model of the role of the NER system in *H. pylori.* ** DNA molecules enter the cytoplasm as ssDNAs. These highly recombinogenic substrates are loaded with RecA filaments which catalyze the invasion of chromosomal DNA whenever homology regions are found [37]. This invasion results in DNA distortions that are recognized by the UvrAB complex. Since UvrC does not seem to be essential for the strand incision, but is involved in the regulation of the import length, another endonuclease might be recruited to generate the incisions (X?). In homology to *E. coli*, UvrB might engage UvrD in order to remove the cut fragment and unwind the DNA. Finally, the nicked region will be repaired by DNA polymerase I and ligase using the donor DNA as template. Early in the process, UvrD competes for the RecA-ssDNA substrates and works as an anti-recombinase by dismantling the RecA filaments leading to strand restoration.

Based on the data from our genetic analysis, we propose a model for homologous recombination in *H. pylori* (Figure 4), where DNA molecules enter the cytoplasm as ssDNAs, which are highly recombinogenic substrates [35,36], and are loaded with RecA as nucleoprotein filaments [37]. Thereafter, RecA catalyzes the duplex invasion whenever homology regions are encountered within the genomic *H. pylori* recipient strain [36]. This results in DNA distortions that are recognized by the UvrAB complex. It remains unclear how strand breaks are introduced after this recognition, since the data indicate that UvrC is either not involved in this process, or can be functionally replaced by a different enzyme with partly redundant function. The helicase UvrD catalyzes the removal of the incised fragment and the unwinding of the DNA. Finally, the incised region will then be repaired by DNA polymerase I and ligase. UvrD also works as an anti-recombinase, by dismantling the RecA-ssDNA complex and thus leading to the restoration of the template, as found previously in *E. coli* and suggested for *H. pylori*[23,26].

## Conclusions

Our study provides evidence for a dual role of the NER system in *H. pylori*: besides its function in safeguarding genome integrity from DNA-damaging agents, it also contributes to its genetic diversity. This is accomplished first by the generation of spontaneous mutations, and second, by controlling import frequency and import length of donor DNA via homologous recombination. Even though the importance of recombination in the genetic variability of *H. pylori* has been well characterized, less is known about the molecular mechanisms and the regulation of the DNA incorporation. Therefore, the investigation of the NER system in homologous recombination and the specific role of UvrC in the regulation of import length are of interest for future studies. Since the gastric habitat of *H. pylori* is likely to be rich in DNA damaging agents, it will be of interest to study the roles of NER components in *H. pylori* genetic diversification under *in vivo* conditions, e.g. in suitable animal models. Finally, the results show the functional versatility of apparently conserved housekeeping proteins such as the NER components, emphasizing the importance of comparative functional analyses in diverse organisms, such as other naturally competent and recombining bacteria.

## Methods

### Bacterial strains and culture conditions

Bacterial strains used in this study are listed in Additional file 4: Table S1. *H. pylori* wild type strains 26695 [21] and J99 [38] were cultured from frozen stocks on blood agar plates (Blood agar base II, Oxoid, Wesel, Germany) containing 10% horse blood and a mix of antibiotics (vancomycin [10 mg/l], polymyxin B [3.2 mg/l], amphotericin B [4 mg/l], and trimethoprim [5 mg/l]). The agar plates were kept in an incubator with 5% O_2_, 10% CO_2_ and 85% N_2_ at 37°C for 24–48 h. Mutant strains were cultivated on blood agar plates containing kanamycin (20 μg/ml), chloramphenicol (20 μg/ml), or both antibiotics as required. Liquid cultures were grown in brain heart infusion (BHI, Oxoid) medium with yeast extract (2.5 g/l), 10% heat inactivated horse serum and an antibiotics cocktail (see above) in microaerobic atmosphere using air-tight jars (Oxoid) and Anaerocult® C gas generating bags (Merck).

For the DNA cloning experiments, we used *E. coli* strains DH5α [39] and MC1061 [40]. These strains were grown in LB broth or on LB plates (Lennox L Broth, Invitrogen GmbH, Karlsruhe, Germany) supplemented with ampicillin (200 μg/ml), chloramphenicol (20 μg/ml) and/or kanamycin (20 μg/ml) as required.

### DNA techniques

All standard procedures (cloning, DNA amplification, purification and manipulation) were performed according to standard protocols [41]. Total genomic bacterial DNA was prepared using the QIAamp DNA Minikit (QIAGEN, Hilden, Germany). Large-scale purification of bacterial chromosomal DNA was performed using QIAGEN Genomic-tip 100/G columns according to the manufacturer’s instructions. Plasmid DNA from *E. coli* strains was isolated using QIAGEN tip 100 columns.

### Insertion mutagenesis in *H. pylori*

The construction of *uvrAuvrBuvrC* and *uvrD* mutants by natural transformation-mediated allelic exchange was performed as described previously [42]. A list of the oligonucleotides used for mutagenesis, including the introduced restriction sites is provided in Additional file 4: Table S2. Briefly, the target genes were amplified by PCR and cloned into pUC18. The resulting plasmids (Additional file 4: Table S3) were used for inverse PCR amplification. Inverse PCR reactions were designed to result in the deletion of a part of the target gene (*uvrAuvrBuvrC*) or the complete gene (*uvrD*), and to introduce a unique BglII (or PstI for the *uvrD* construct) restriction site. The PCR products were subsequently digested with BglII (or PstI), and ligated with a kanamycin or chloramphenicol resistance cassette (*aphA-3* or *cat*; [43,44] flanked by the compatible BamHI (or BglII) restriction sites. The direction of transcription of the antibiotic resistance genes (kanamycin [Km] and chloramphenicol [Cm]) was the same as that of the target gene to avoid possible polar effects.

Plasmids containing the interrupted gene were used as suicide plasmids for natural transformations of the *H. pylori* strain 26695. The successful chromosomal replacement of the target gene with the disrupted gene construct via allelic exchange (double crossover) was checked by PCR using suitable primer combinations.

### Functional complementation of mutants

Functional complementation experiments for the *uvrB* and *uvrC* mutant strains were performed by inserting an intact copy of the target gene into the *ureAB* locus (Additional file 4: Table S3). To do so, the ORFs HP1114 and HP0821 were cloned in the pADC vector [45] downstream of the strong *ureAB* promoter, creating the plasmids pSUS2646 and pSUS2644 (Additional file 4: Table S2 and S3). Functional complementation of *uvrA* was performed by inserting an intact copy of the *uvrA* gene together with 400 bp of DNA upstream of the start codon containing the putative *uvrA* promoter into the *rdxA* locus. The ORF HP0705 plus the upstream region were cloned in the pCJ535 vector, creating the plasmid pSUS3009. These suicide plasmids were introduced via natural transformation into the single gene mutant strains 26695 *uvrA*, 26695 *uvrB*, and 26695 *uvrC*, and the transformants were selected on Km/Cm blood agar plates. The correct insertion of the complementing genes in the *ureAB* or *rdxA* locus was controlled by PCR and sequence analysis of the insertion sites.

### *In vitro* transformation system of *H. pylori*, determination of mutation and recombination frequencies and import sizes

The transformation system used to quantitate, in parallel, mutation and recombination rates as well as the length of the DNA fragments incorporated into the chromosome after recombination has been described previously [12]. Mutation rates obtained with this system have been shown to be in excellent agreement with fluctuation analysis [42]. From each experiment, at least 16 clones were expanded in order to sequence a fragment (1663 bp) of the *rpoB* gene (see below). The experiments were reproduced three times for each *H. pylori* mutant strain. To determine the length of import events in the *rpoB* gene, a 2361 bp PCR fragment of *rpoB* was amplified with primers HPrpoB-1 and HPrpoB-6 as previously described [12] and Additional file 4: Table S2). This PCR product was used as template for the sequencing reactions with the primers HPrpoB-3, -4, -5, -6, -9w, and −10. The six sequences from each rifampicin resistant clone were assembled using the software Bionumerics V 4.5 (Applied Maths, Sint-Martens-Latem, Belgium), yielding a continuous, double-stranded 1663 bp fragment of *rpoB* that included the Rif resistance-mediating point mutation of the donor strain.

### PCR-based prescreening for clones with DNA imports in strain 26695 *uvrA*

Due to the low recombination frequency in 26695 *uvrA*, it was necessary to screen the Rif resistant clones after transformation in order to distinguish recombinants from spontaneous mutants. This was accomplished by allele-specific PCR using the primers HPrpoB-IscrX and HPrpoB-4, which specifically detect the Rif resistance mediating point mutation in strain J99-R3 [12,46]. PCR positive clones were used for sequencing as described above.

### UV irradiation of mutant strains

Bacteria were cultured on blood agar plates for 24 h as described above. Cells were then suspended in phosphate buffered saline (PBS) and appropriate dilutions to obtain ~100, 500 and 1,000 colonies were plated on blood agar plates in two triplicate batches. As a control, the first batch was not exposed to UV light to obtain the total cell number. The plates of the second batch were placed under a UV-C lamp (OSRAM HNS 30 W OFR, wavelength 254 nm) for two seconds at a distance of 40 cm, corresponding to approximately 100 J/m^2^. All plates were incubated for 72 h as previously described, colonies were counted and the percentage of surviving cells was calculated.

### Growth properties of *H. pylori* strains

Growth curves were monitored in liquid cultures (BHI broth including 10% horse serum and antibiotics). Strains were grown for <24 h on blood agar plates and then harvested in BHI broth. The OD_600_ of the suspension was measured and diluted to a starting concentration of 2.1 × 10^7^ bacteria/ml. Cultures were then incubated at 37°C in a rotary shaker (175 rpm) under microaerobic conditions. The optical density was measured at regular intervals.

### Statistical methodology

Statistical analysis was performed using Bayesian model comparison, where two competing hypotheses are weighted against each other by computing the ratio of probabilities of the observed data under the two hypotheses. This ratio is called a Bayes Factor (see refs. [47,48] for reviews). A benefit of this approach is that it accounts for the relative complexity of the hypotheses, so that the more complex one is validated only if the data justifies it. Interpretation of the Bayes Factor was done following the scale of Jeffreys [49]: Negative (<1); Barely worth mentioning (1–3); Substantial (3–10); Strong (10–30); Very strong (30–100); Decisive (>100).

When the Bayes Factor could not be analytically computed, the Bayes Information Criterion (BIC; refs. [47,50] was used as an estimate:

(1) $$BF\approx \exp\left(\frac{BIC_{1\;}-BIC_{2}}{2}\right)=\exp\left(l_{2\;}-l_{1\;}+\text{log}(n)\frac{k_{1\;}-k_{2}}{2}\right)$$

where *l*_1_ and *l*_2_ are the maximized value of the log-likelihood under the two models, *k*_1_ and *k*_2_ the number of parameters in the two models, and n the number of observations. Comparisons of frequency data between any two recipient/donor combinations were done using the BIC with one hypothesis being that the data from the two combinations comes from the same Normal distribution and the other hypothesis being that they come from two distinct Normal distributions.

Recombination start or end point were not observed exactly, but instead in an interval [m_i_;M_i_] (with M_i_ = ∞ if the beginning or end is out of the sequenced region). We assume a geometric length of recombination with mean δ on both sides of the mutation conferring resistance to rifampicin. Model comparisons using the BIC found no evidence for a difference between the lengths on the two sides, and no support for a more complex negative binomial distribution which has an additional parameter compared to the geometric distribution. The likelihood of N observations is therefore equal to:

(2) $$L\left(\delta \right)=\prod_{i=1}^{N}\left({\left(1-\frac{1}{\delta }\right)}^{m_{i\;}}-{\left(1-\frac{1}{\delta }\right)}^{m_{i}}\right)$$

The effect of gene knock-outs on the lengths of import was evaluated using the BIC where one hypothesis is that δ remains the same and the other hypothesis is that δ changes.

Let *p* denote the probability of occurrence of ISR in a clone. The number *m* of clones containing ISR amongst *n* clones is thus distributed as Binomial(*n*,*p*). A Jeffrey's prior was assumed on *p* (i.e. Beta (½,½)). We assessed whether the probability of ISR was identical between two recipient/donor combinations (*m*_1_,*n*_1_ and *m*_2_,*n*_2_) using the Bayes Factor:

(3) $$BF=\frac{B(m_{1\;}+1/2,n_{1}-m_{1\;}+m_{1\;}+1/2)\;B\left(m_{2\;}+1/2,n_{2\;}-m_{2\;}+1/2\right)}{B\left(m_{2\;}+m_{2\;}+1/2,n_{1\;}+n_{2\;}-m_{1\;}-m_{2\;}+1/2\right)}$$

where B(.,.) denotes the Euler Beta function.

## Competing interests

The authors declare to have no competing interest.

## Authors’ contributions

CM, JK, SK, CK, CB and SS designed the research, CM, JK, SK, CK and CB performed the experiments. XD performed all statistical analyses. CM, JK, XD, CB and SS wrote the paper. All authors analyzed data and saw and approved the paper.

## Supplementary Material

Additional file 1 **Figure S1.** Growth curves (OD_600_) of *H. pylori* strains 26695, 26695*uvrA*, 26695*uvrB*, 26695*uvrC*, 26695*uvrD* and complemented mutant strains. Click here for file

Additional file 2 **Figure S2.** Nucleotide sequence alignment of the 1663 bp fragment of the *rpoB* gene used to determine import length. Sequences are shown for strains 26695, J99 and J99R3. The sequences were aligned using CLC Sequence Viewer v6.6.1 and the point mutation (A1618T) that confers Rif resistance is labeled. Click here for file

Additional file 3 **Figure S3.** Amino acid sequence alignments of the four NER components, UvrA, UvrB, UvrC and UvrD. The primary sequences from *H. pylori* 26695, *C. jejuni* NCTC11168, *E. coli* K12 and *S. aureus* N315 were aligned by performing a muscle alignment [51] using CLC Sequence Viewer v6.6.1. **A.** UvrA (*H. pylori* 26695 HP0705, *C. jejuni* NCTC11168 Cj0342c, *E. coli* K12 EG11061 and *S. aureus* N315 SA0714). **B.** UvrB (*H. pylori* 26695 HP1114, *C. jejuni* NCTC11168 Cj0680c, *E. coli* K12 EG11062 and *S. aureus* N315 SA0713. **C.** UvrC (*H. pylori* 26695 HP0821, *C. jejuni* NCTC11168 Cj1246c, *E. coli* K12 EG11063 and *S. aureus* N315 SA0993). **D.** UvrD (*H. pylori* 26695 HP1478, *C. jejuni* NCTC11168 Cj1101, *E. coli* K12 EG11064 and *S. aureus* N315 SA1721). The UvrD equivalent protein in Gram positive bacteria is known as PcrA. Amino acids conserved in three or all four orthologs are labelled with light or dark blue shading, respectively. Click here for file

Additional file 4 **Table S1.** Bacterial strains [12,21,39,40]. **Table S2**. Oligonucleotide primers and PCR products used in this study [12,44]. **Table S3**. Plasmids used in this study [12,23,43-45,52]. Click here for file
