# Supplementary figures and images for: The nucleotide excision repair (NER) system of Helicobacter pylori: Role in mutation prevention and chromosomal import patterns after natural transformation
Source: BMC Microbiol. 2012 May 6;12:67. doi: 10.1186/1471-2180-12-67 (PMC3438104; doi:10.1186/1471-2180-12-67)

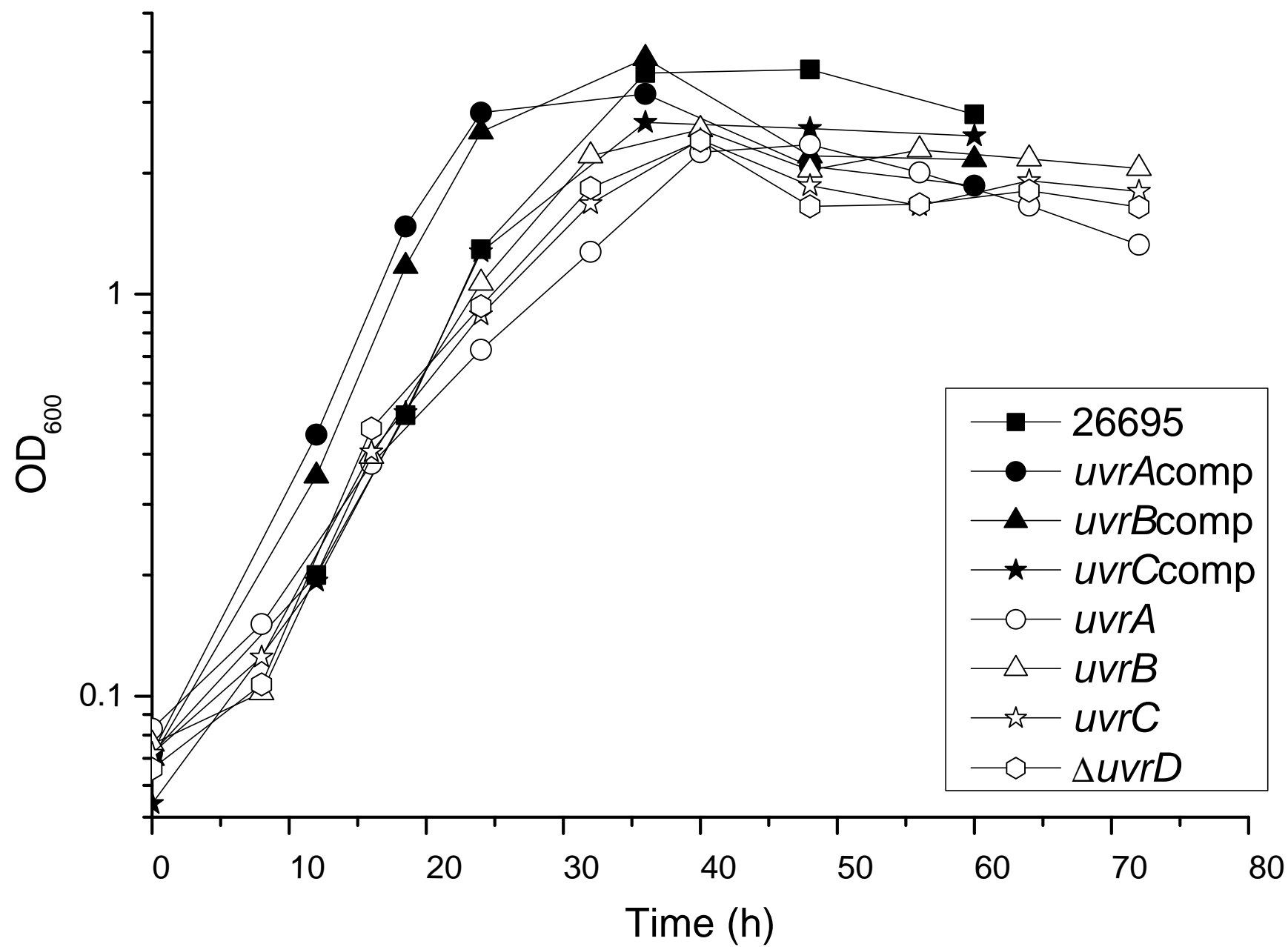

Supplement: Additional file 1 — Figure S1. Growth curves (OD600) of H. pylori strains 26695, 26695uvrA, 26695uvrB, 26695uvrC, 26695uvrD and complemented mutant strains. [file 1471-2180-12-67-S1.pdf]
