# Supplementary material for: The nucleotide excision repair (NER) system of Helicobacter pylori: Role in mutation prevention and chromosomal import patterns after natural transformation
Source: BMC Microbiol. 2012 May 6;12:67. doi: 10.1186/1471-2180-12-67 (PMC3438104; doi:10.1186/1471-2180-12-67)
Supplement: Additional file 2 — Figure S2. Nucleotide sequence alignment of the 1663 bp fragment of the rpoB gene used to determine import length. Sequences are shown for strains 26695, J99 and J99R3. The sequences were aligned using CLC Sequence Viewer v6.6.1 and the point mutation (A1618T) that confers Rif resistance is labeled. [file 1471-2180-12-67-S2.pdf]

```
        760                 780                 800                 820                 840
26695 AGAATGGAATTTGACTTGAAAGATCCTCAAGGCAAGGTTATTCTTTTAGCGGGTAAAAAGCTCACTTCAAGAAAGATTAAAGAGCTTAAAGAAAACCA 98
J99   AGAATGGAATTTGACTTGAAAGACCCGCAAGGCAAGATTATCCTTTTGGCGGGTAAAAAGCTCACTTCAAGGAAAATTAAAGAACTTAAAGAAAACCA 98
J99R3 AGAATGGAATTTGACTTGAAAGACCCGCAAGGCAAGATTATCCTTTTGGCGGGTAAAAAGCTCACTTCAAGGAAAATTAAAGAACTTAAAGAAAACCA 98
        860                 880                 900                 920                 940
26695 TTTAGAATGGGTGGAATACCCTATGGATATTTTACTCAATCGCCATTTAGCTGAGCCTGTTATGGTAGGGAAAGAAGTCTTATTGGACATGCTCACTC 196
J99   TTTAGAATGGGTGGAATACCCTATGGATATTTTACTCAATCGTCATTTAGCTGAGCCTGTTATGGTAGGGAAAGAAGTTTTATTGGACATGCTCACAC 196
J99R3 TTTAGAATGGGTGGAATACCCTATGGATATTTTACTCAATCGTCATTTAGCTGAGCCTGTTATGGTAGGGAAAGAAGTTTTATTGGACATGCTCACAC 196
        960                 980                 1.000               1.020               1.040
26695 AGCTAGATAAAAACAAATTAGAAAAAATCCACGATTTAGGCGTGCAAGAATTTGTGATCATCAACGATCTGGCGTTAGGGCATGACGCTTCCATTATC 294
J99   AGTTGGATAAAAACAGACTAGAAAAAATCCACGATCTAGGCGTGCAAGAATTTGTGATCATCAACGATTTAGCGTTAGGGCATGACGCTTCCATTATC 294
J99R3 AGTTGGATAAAAACAGACTAGAAAAAATCCACGATCTAGGCGTGCAAGAATTTGTGATCATCAACGATTTAGCGTTAGGGCATGACGCTTCCATTATC 294
        1.060               1.080               1.100               1.120               1.140
26695 CAATCTTTTTCAGCCGATTCTGAGTCTTTGAAATTACTCAAGCAAACCGAAAAAATTGATGATGAAAACGCTCTAGCGGCGATTCGTATCCATAAGGT 392
J99   CATTCTTTCTCAGCCGATCATGAGTCTTTGAAATTGCTCAAGCAAACTGAAAAGATTGATGATGAAAACGCTCTAGCGGCGATTCGTATCCATAAGGT 392
J99R3 CATTCTTTCTCAGCCGATCATGAGTCTTTGAAATTGCTCAAGCAAACTGAAAAGATTGATGATGAAAACGCTCTAGCGGCGATTCGTATCCATAAGGT 392
        1.160               1.180               1.200               1.220               1.240
26695 TATGAAACCAGGCGATCCCGTTACGACTGAAGTGGCTAAGCAGTTTGTCAAAAAACTTTTCTTTGATTCCAGAACGCTATGATTTGACCATGGTGGGCC 490
J99   TATGAAACCAGGTGATCCTGTTACGACTGAAGTGGCTAAGCAATTTGTTAAGAAACTTTTCTTTGACCCTGAACGCTATGATTTGACCATGGTGGGCC 490
J99R3 TATGAAACCAGGTGATCCTGTTACGACTGAAGTGGCTAAGCAATTTGTTAAGAAACTTTTCTTTGACCCTGAACGCTATGATTTGACCATGGTGGGCC 490
        1.260               1.280               1.300               1.320
26695 GCATGAAAATGAATCACAAGTTAGGCTTGCATGTGCCTGATTACATTACGACTTTAACGCATGAAGATATTATCACCACCGTTAAATACCTCATGAAG 588
J99   GTATGAAAATGAATCACAAGTTAGGCTTGCATGTGCCTGATTACATCACGACTTTAACGCATGAAGATATTATCACCACCGTTAAATACCTCATGAAG 588
J99R3 GTATGAAAATGAATCACAAGTTAGGCTTGCATGTGCCTGATTACATCACGACTTTAACGCATGAAGATATTATCACCACCGTTAAATACCTCATGAAG 588
        1.340               1.360               1.380               1.400               1.420
26695 ATCAAAAACAATCAAGGCAAGATTGATGACCAGGGACCACTTGGGCAATCGTAGGATTAGGCGGTAGGGGAATTGTTGGCCAATGAATTGCATTCAGG 686
J99   ATCAAAAACAATCAGGGCAAGATTGATGATAGGGATCACTTAGGCAATCGCAGGATCAGAGCCGTTGGGGAACTATTGGCCAATGAATTGCATTCAGG 686
J99R3 ATCAAAAACAATCAGGGCAAGATTGATGATAGGGATCACTTAGGCAATCGCAGGATCAGAGCCGTTGGGGAACTATTGGCCAATGAATTGCATTCAGG 686
        1.440               1.460               1.480               1.500               1.520
26695 TTTAGTGAAAATGCAAAAGACCATTAAAGACAAGCTCACTACCATGAGCGGGGCTTTTGATTCGCTCATGCCCCATGACTTGGTCAATTCTAAAATGA 784
J99   CTTAGTGAAAATGCAAAAGACCATTAAAGACAAACTCACCACCATGAGCGGGGCTTTTGATTCGCTCATGCCCCATGATTTAGTCAATTCTAAAATGA 784
J99R3 CTTAGTGAAAATGCAAAAGACCATTAAAGACAAACTCACCACCATGAGCGGGGCTTTTGATTCGCTCATGCCCCATGATTTAGTCAATTCTAAAATGA 784
        1.540               1.560               1.580               1.600               1.620
26695 TCACAAGCACCATCATGGAATTTTTCATGGGCGGTCAGCTCTCGCAATTTATGGATCAAACGAATCCCTTGAGTGAGGTTACGCACAAGCGCCGCCTT 882
J99   TCACCAGCACCATCATGGAATTTTTCATGGGCGGTCAGCTCTCGCAATTCATGGATCAAACCAACCCTTTGAGTGAAGTTACGCACAAACGACGCCTT 882
J99R3 TCACCAGCACCATCATGGAATTTTTCATGGGCGGTCAGCTCTCGCAATTCATGGATCAAACCAACCCTTTGAGTGAAGTTACGTACAAACGACGCCTT 882
        1.640               1.660               1.680               1.700               1.720
26695 TCAGCGCTCGGCGAAGGGGGGTTGGTGAAAGACAGAGTGGGGTTTGAAGCCAGGGATGTGCACCCCACGCATTATGGCCGAATTTGTCCCATTGAGAC 980
J99   TCAGCGCTCGGTGAAGGGGGGTTAGTCAAAGACAGGGTAGGGTTTGAAGCCAGGGATGTGCACCCCACGCATTATGGCCGAATTTGTCCCATTGAAAC 980
J99R3 TCAGCGCTCGGTGAAGGGGGGTTAGTCAAAGACAGGGTAGGGTTTGAAGCCAGGGATGTGCACCCCACGCATTATGGCCGAATTTGTCCCATTGAAAC 980
        1.740               1.760               1.780               1.800               1.820
26695 CCCAGAAGGTCAAAATATCGGTCTGATCAACACCCTTTCCACTTTCACAAGAGTGAATGATTTAGGCTTTATTGAAGCCCCTTATAAAAAGGTTGTGG 1078
J99   CCCAGAAGGTCAAAATATCGGTTTGATCAACACCCTTTCCACTTTCACAAGAGTGAATGATTTAGGCTTTATTGAAGCCCCTTATAAAAAGGTTGTGG 1078
J99R3 CCCAGAAGGTCAAAATATCGGTTTGATCAACACCCTTTCCACTTTCACAAGAGTGAATGATTTAGGCTTTATTGAAGCCCCTTATAAAAAGGTTGTGG 1078
        1.840               1.860               1.880               1.900               1.920
26695 ATGGCAAGGTCGTGGGTGAGACGATTTATTTGACCGCTATTCAAGAAGACAGCCACATCATCGCTCCCGCAAGCACCCCCATTGATGAAGAGGGGAAT 1176
J99   ATGGCAAGGTGGCGGGCGAGACGATTTATTTGACCGCTATTCAAGAAGACAGCCACATCATCGCTCCCGCAAGCACCCCCATTGATGAAGAAGGGAAT 1176
J99R3 ATGGCAAGGTGGCGGGCGAGACGATTTATTTGACCGCTATTCAAGAAGACAGCCACATCATCGCTCCCGCAAGCACCCCCATTGATGAAGAAGGGAAT 1176
        1.940               1.960               1.980               2.000               2.020
26695 ATTTTGGGCGATTTGATTGAAACGCGCGTGGAAGGCGAGATCGTTTTAAACGAAAAAAGCAAAGTAACCTTAATGGATTTAAGCTCTAGCATGCTAGT 1274
J99   ATTTTAGGCGATTTGATTGAAACGCGAGTGGAAGGCGAGATCGTTTTAAACGAAAAGAGTAAAGTTACCTTAATGGATCTAAGCTCTAGCATGCTAGT 1274
J99R3 ATTTTAGGCGATTTGATTGAAACGCGAGTGGAAGGCGAGATCGTTTTAAACGAAAAGAGTAAAGTTACCTTAATGGATCTAAGCTCTAGCATGCTAGT 1274
        2.040               2.060               2.080               2.100               2.120
26695 GGGGGTAGCCGCATCGCTCATTCCTTTCTTAGAGCATGATGACGCCAACCGTGCCTTAATGGGGACTAACATGCAGCGCCAAGCGGTGCCCTTATTAA 1372
J99   AGGGGTAGCCGCATCGCTCATTCCTTTCTTAGAGCATGATGACGCTAACCGCGCTCTAATGGGGACTAACATGCAGCGCCAAGCCGTGCCTTTATTAA 1372
J99R3 AGGGGTAGCCGCATCGCTCATTCCTTTCTTAGAGCATGATGACGCTAACCGCGCTCTAATGGGGACTAACATGCAGCGCCAAGCCGTGCCTTTATTAA 1372
        2.140               2.160               2.180               2.200               2.220
26695 GAAGCGACGCTCCCATTGTAGGCACGGGGATTGAAAAAATTATTGCTAGGGATTCTTGGGGAGCGATCAAAGCCAATCGCGCAGGCGTTGTAGAAAAA 1470
J99   GAAGCGACGCCCCCATTGTAGGCACAGGGATTGAAAAAATTATCGCTAGGGATTCTTGGGGAGCGATCAAGGCCAATCGCGCAGGCGTTGTAGAAAAA 1470
J99R3 GAAGCGACGCCCCCATTGTAGGCACAGGGATTGAAAAAATTATCGCTAGGGATTCTTGGGGAGCGATCAAGGCCAATCGCGCAGGCGTTGTAGAAAAA 1470
        2.240               2.260               2.280               2.300
26695 ATTGATTCTAAAAATATTTATATTTTAGGCGAAAGCAAAGAAGAAGCCTATATTGATGCGTATTCTTTGCAAAAAAACTTGCGCACCAACCAAAACAC 1568
J99   ATTGATTCTAAAAACATTTACATTTTAGGCGAAGGCAAAGAAGAAGCCTATATTGACGCGTATTCTTTGCAAAAAAACTTGCGCACCAACCAAAACAC 1568
J99R3 ATTGATTCTAAAAACATTTACATTTTAGGCGAAGGCAAAGAAGAAGCCTATATTGACGCGTATTCTTTGCAAAAAAACTTGCGCACCAACCAAAACAC 1568
        2.320               2.340               2.360               2.380               2.400
26695 CAGTTTCAATCAAGTCCCTATCGTTAAAGTGGGCGATAAAGTGGGAGCCGGGCAAATCATCGCTGATGGCCCTAGCATGGATAGAGGCGAGTTAG 1663
J99   CAGTTTCAATCAAGTCCCTATCGTTAAAGTGGGCGATAAAGTGGAAGCCGGGCAAATCATCGCTGATGGCCCTAGCATGGATAGGGGCGAGTTAG 1663
J99R3 CAGTTTCAATCAAGTCCCTATCGTTAAAGTGGGCGATAAAGTGGAAGCCGGGCAAATCATCGCTGATGGCCCTAGCATGGATAGGGGCGAGTTAG 1663
```
