# Supplementary material for: The nucleotide excision repair (NER) system of Helicobacter pylori: Role in mutation prevention and chromosomal import patterns after natural transformation
Source: BMC Microbiol. 2012 May 6;12:67. doi: 10.1186/1471-2180-12-67 (PMC3438104; doi:10.1186/1471-2180-12-67)
Supplement: Additional file 3 — Figure S3. Amino acid sequence alignments of the four NER components, UvrA, UvrB, UvrC and UvrD. The primary sequences from H. pylori 26695, C. jejuni NCTC11168, E. coli K12 and S. aureus N315 were aligned by performing a muscle alignment [51] using CLC Sequence Viewer v6.6.1. A. UvrA (H. pylori 26695 HP0705, C. jejuni NCTC11168 Cj0342c, E. coli K12 EG11061 and S. aureus N315 SA0714). B. UvrB (H. pylori 26695 HP1114, C. jejuni NCTC11168 Cj0680c, E. coli K12 EG11062 and S. aureus N315 SA0713. C. UvrC (H. pylori 26695 HP0821, C. jejuni NCTC11168 Cj1246c, E. coli K12 EG11063 and S. aureus N315 SA0993). D. UvrD (H. pylori 26695 HP1478, C. jejuni NCTC11168 Cj1101, E. coli K12 EG11064 and S. aureus N315 SA1721). The UvrD equivalent protein in Gram positive bacteria is known as PcrA. Amino acids conserved in three or all four orthologs are labelled with light or dark blue shading, respectively. [file 1471-2180-12-67-S3.pdf]

A

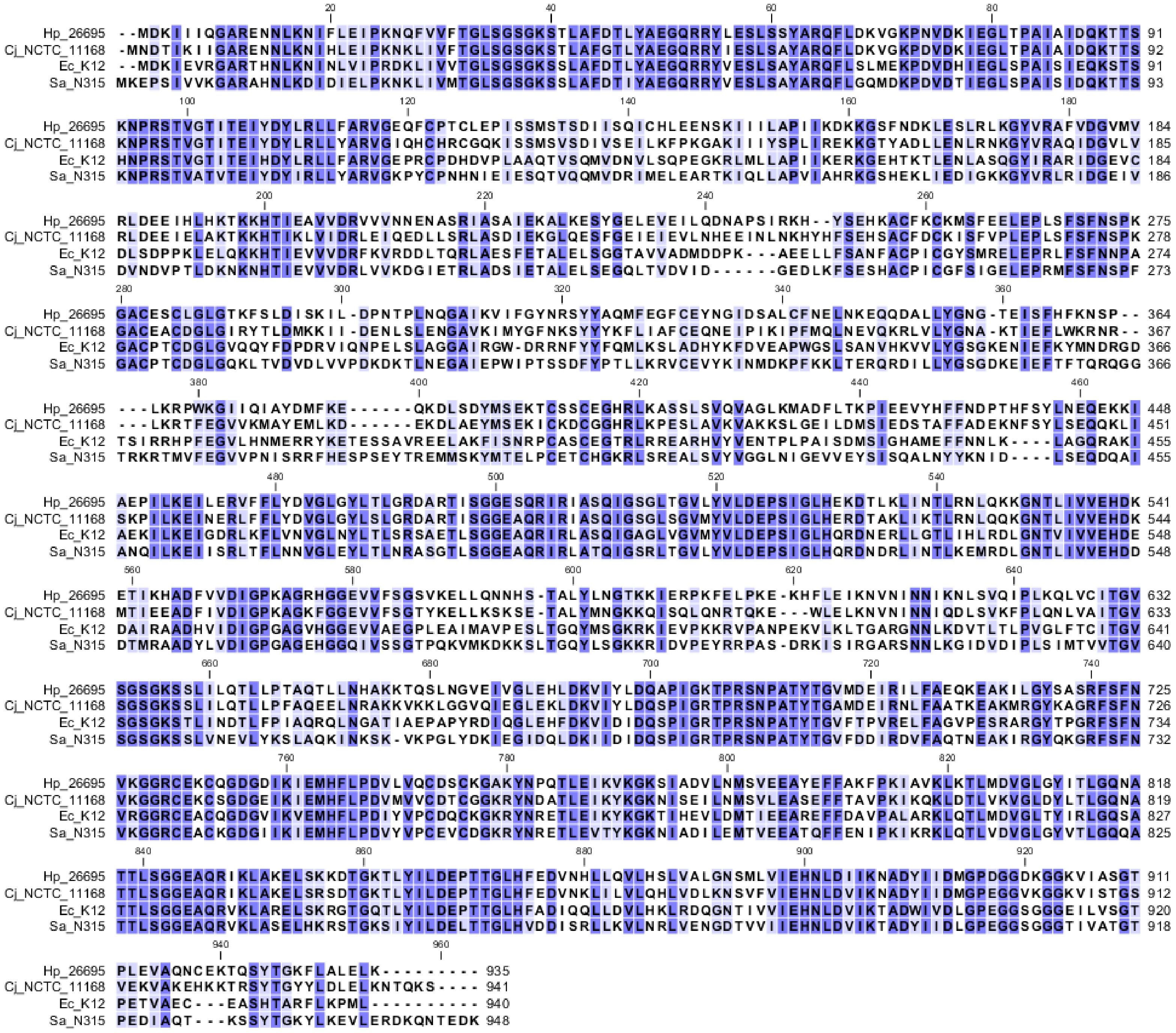

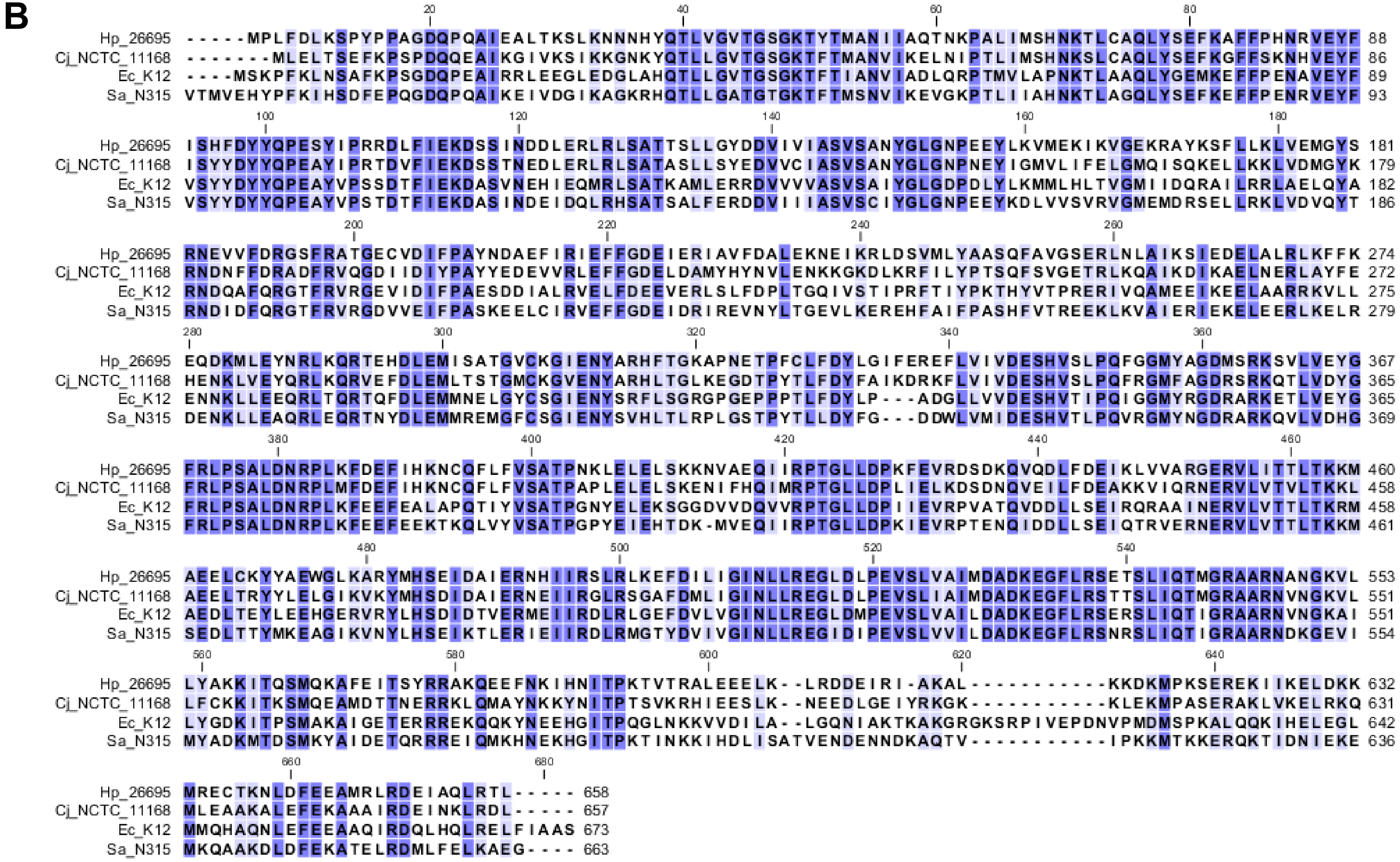
B
20 40 60 80
Hp_26695 -----MPLFDLKSPYPPAGDQPQAIEALTKSLKNNNHYQTLVGVTGSGKTYTMANIIAQTNKPALIMSHNKTLCAQLYSEFKAFFPHNRVEYF 88
Cj_NCTC_11168 -------MLELTSEFKPSPDQQEAIKGIVKSIKKGNKYQTLLGVTGSGKTFTMANVIKELNIPTLIMSHNKSLCAQLYSEFKGFFSKNHVEYF 86
Ec_K12 ----MSKPFKLNSAFKPSGDQPEAIRRLEEGLEDGLAHQTLLGVTGSGKTFTIANVIADLQRPTMVLAPNKTLAAQLYGEMKEFFPENAVEYF 89
Sa_N315 VTMVEHYPFKIHSDFEPQGDQPQAIKEIVDGIKAGKRHQTLLGATGTGKTFTMSNVIKEVGKPTLIIAHNKTLAGQLYSEFKEFFPENRVEYF 93
100 120 140 160 180
Hp_26695 ISHFDYYQPESYIPRRDLFIEKDSSINDDLERLRLSATTSLLGYDDVIVIASVSANYGLGNPEEYLKVMEKIKVGEKRAYKSFLLKLVEMGYS 181
Cj_NCTC_11168 ISYYDYYQPEAYIPRTDVFIEKDSSTNEDLERLRLSATASLLSYEDVVCIASVSANYGLGNPNEYIGMVLIFELGMQISQKELLKKLVDMGYK 179
Ec_K12 VSYYDYYQPEAYVPSSDTFIEKDASVNEHIEQMRLSATKAMLERRDVVVVASVSAIYGLGDPDLYLKMMLHLTVGMIIDQRAILRRLAELQYA 182
Sa_N315 VSYYDYYQPEAYVPSTDTFIEKDASINDEIDQLRHSATSALFERDDVIIIASVSCIYGLGNPEEYKDLVVSVRVGMEMDRSELLRKLVDVQYT 186
200 220 240 260
Hp_26695 RNEVVFDRGSFRATGECVDIFPAYNDAEFIRIEFFGDEIERIAVFDALEKNEIKRLDSVMLYAASQFAVGSERLNLAIKSIEDELALRLKFFK 274
Cj_NCTC_11168 RNDNFFDRADFRVQGDIIDIYPAYYEDEVVRLEFFGDELDAMYHYNVLENKKGKDLKRFILYPTSQFSVGETRLKQAIKDIKAELNERLAYFE 272
Ec_K12 RNDQAFQRGTFRVRGEVIDIFPAESDDIALRVELFDEEVERLSLFDPLTGQIVSTIPRFTIYPKTHYVTPRERIVQAMEEIKEELAARRKVLL 275
Sa_N315 RNDIDFQRGTFRVRGDVVEIFPASKEELCIRVEFFGDEIDRIREVNYLTGEVLKEREHFAIFPASHFVTREEKLKVAIERIEKELEERLKELR 279
280 300 320 340 360
Hp_26695 EQDKMLEYNRLKQRTEHDLEMISATGVCKGIENYARHFTGKAPNETPFCLFDYLGIFEREFLVIVDESHVSLPQFGGMYAGDMSRKSVLVEYG 367
Cj_NCTC_11168 HENKLVEYQRLKQRVEFDLEMLTSTGMCKGVENYARHLTGLKEGDTPYTLFDYFAIKDRKFLVIVDESHVSLPQFRGMFAGDRSRKQTLVDYG 365
Ec_K12 ENNKLLEEQRLTQRTQFDLEMMNELGYCSGIENYSRFLSGRGPGEPPPTLFDYLP---ADGLLVVDESHVTIPQIGGMYRGDRARKETLVEYG 365
Sa_N315 DENKLLEAQRLEQRTNYDLEMMREMGFCSGIENYSVHLTLRPLGSTPYTLLDYFG---DDWLVMIDESHVTLPQVRGMYNGDRARKQVLVDHG 369
380 400 420 440 460
Hp_26695 FRLPSALDNRPLKFDEFIHKNCQFLFVSATPNKLELELSKKNVAEQIIRPTGLLDPKFEVRDSDKQVQDLFDEIKLVVARGERVLITTLTKKM 460
Cj_NCTC_11168 FRLPSALDNRPLMFDEFIHKNCQFLFVSATPAPLELELSKENIFHQIMRPTGLLDPLIELKDSDNQVEILFDEAKKVIQRNERVLVTVLTKKL 458
Ec_K12 FRLPSALDNRPLKFEEFEALAPQTIYVSATPGNYELEKSGGDVVDQVVRPTGLLDPIIEVRPVATQVDDLLSEIRQRAAINERVLVTTLTKRM 458
Sa_N315 FRLPSALDNRPLKFEEFEEKTKQLVYVSATPGPYEIEHTDK-MVEQIIRPTGLLDPKIEVRPTENQIDDLLSEIQTRVERNERVLVTTLTKKM 461
480 500 520 540
Hp_26695 AEELCKYYAEWGLKARYMHSEIDAIERNHIIRSLRLKEFDILIGINLLREGLDLPEVSLVAIMDADKEGFLRSETSLIQTMGRAARNANGKVL 553
Cj_NCTC_11168 AEELTRYYLELGIKVKYMHSDIDAIERNEIIRGLRSGAFDMLIGINLLREGLDLPEVSLIAIMDADKEGFLRSTTSLIQTMGRAARNVNGKVL 551
Ec_K12 AEDLTEYLEEHGERVRYLHSDIDTVERMEIIRDLRLGEFDVLVGINLLREGLDMPEVSLVAILDADKEGFLRSERSLIQTIGRAARNVNGKAI 551
Sa_N315 SEDLTTYMKEAGIKVNYLHSEIKTLERIEIIRDLRMGTYDVIVGINLLREGIDIPEVSLVVILDADKEGFLRSNRSLIQTIGRAARNDKGEVI 554
560 580 600 620 640
Hp_26695 LYAKKITQSMQKAFEITSYRRAKQEEFNKIHNITPKTVTRALEEELK--LRDDEIRI-AKAL-----------KKDKMPKSEREKIIKELDKK 632
Cj_NCTC_11168 LFCKKITKSMQEAMDTTNERRKLQMAYNKKYNITPTSVKRHIEESLK--NEEDLGEIYRKGK-----------KLEKMPASERAKLVKELRKQ 631
Ec_K12 LYGDKITPSMAKAIGETERRREKQQKYNEEHGITPQGLNKKVVDILA--LGQNIAKTKAKGRGKSRPIVEPDNVPMDMSPKALQQKIHELEGL 642
Sa_N315 MYADKMTDSMKYAIDETQRRREIQMKHNEKHGITPKTINKKIHDLISATVENDENNDKAQTV-----------IPKKMTKKERQKTIDNIEKE 636
660 680
Hp_26695 MRECTKNLDFEEAMRLRDEIAQLRTL----- 658
Cj_NCTC_11168 MLEAAKALEFEKAAAIRDEINKLRDL----- 657
Ec_K12 MMQHAQNLEFEEAAQIRDQLHQLRELFIAAS 673
Sa_N315 MKQAAKDLDFEKATELRDMLFELKAEG---- 663

C

```
               20                  40                  60                  80
Hp_26695      ----MADLLSSLKNLPNSSGVYQYFDKNRQLLYIGKAKNLKKRIKSYFSIRNNEITPNHRASLRIQMMVKQIAFLETILVENEQDALILENSL 89
Cj_NCTC_11168 LT--KENLENELKTLPNSTGVYQYFNQEGKLLYVGKAKNLKNRVRSYFAFTPN-LHANPRNSLRIQKMIEETVHLEFIATNSEADALILENSF 90
Ec_K12        VSD-QFDAKAFLKTVTSQPGVYRMYDAGGTVIYVGKAKDLKKRLSSYFRSNLA--------SRKTEALVAQIQQIDVTVTHTETEALLLEHNY 84
Sa_N315       LEDYKQRIKNKLNVVPMEPGCYLMKDRNDQVIYVGKAKKLRNRLRSYFTGAHD---------AKTTRLVGEIRRFEFIVTSSETESLLLELNL 84

               100                 120                 140                 160                 180
Hp_26695      IKQLKPKYNILLRDDKTYPYIYMDFSTDFPIPLITRKILKQPGVKYFGPFTSG--AKDILDSLYELLPLVQKKNCIK--DKKACIFYQIERCK 178
Cj_NCTC_11168 IKQLHPKYNILLRDDKTYPYIYVDFEEEFPRFEITRKLVKKSKIKYFGPFFKG--ARELLDALYLYYPLKQKASC-----KSPCIFYQISRCL 176
Ec_K12        IKLYQPRYNVLLRDDKSYPFIFLS-GDTHPRLAMHRG-AKHAKGEYFGPFPNGYAVRETLALLQKIFPIRQCENSVYRNRSRPCLQYQIGRCL 175
Sa_N315       IKQYQPRYNILLKDDKSYPFIKIT-KEKYPRLLVTRT-VKQGTGKYFGPYPNAYSAQETKKLLDRIYPYRKCDKM----PDKLCLYYHIGQCL 171

               200                 220                 240                 260
Hp_26695      APC-ENKITKEEYLKIAKECLEMIENKDR-LIKELELKMERLSNNLRFEEALIYRDRIAKIQKIAPFTCMDLAKLYDLDIFAFYGASNKAVLV 269
Cj_NCTC_11168 APC-DKLISREKYLEILDEAIHALLNPSV-LLKNLEKQMLVLAQNENYEEAAKVRDQIAMIKDLEVKVEIDIAKLEDFEVFALAFKNSVLSTL 267
Ec_K12        GPCVEGLVSEEEYAQQVEYVRLFLSGKDDQVLTQLISRMETASQNLEFEEAARIRDQIQAVRRVTEKQFVSNTG-DDLDVIGVAFDAGMACVH 267
Sa_N315       GPC-VYDVDLSKYAQMTKEITDFLNGEDKTILKSLEERMLTASESLDFERAKEYRDLIQHIQNLTNKQKIMSSDKTIRDVFGYCVDKGWMCIQ 263

               280                 300                 320                 340                 360
Hp_26695      KMFMRGGKIIS--SAFEKIHSLNGFDTDEAMKQAIINHY--QSHLPLMPEQILLNACSNETLKELQEFISHQYSKKIALSIPKKGDKLALIEI 358
Cj_NCTC_11168 RFVVQNGKIISANSKITPIKNDIQWDQNEIYKQLILENF--SMDIPLLANVIYVYEEFEDRVL-LEEILSQRFDKKISIKIPKIGEKRRICDL 357
Ec_K12        VLFIRQGKVLGSRSYFPKVPG--GTELSEVVETFVGQFYLQGSQMRTLPGEILLDFNLSDKTL-LADSLSELAGRKINVQTKPRGDRARYLKL 357
Sa_N315       VFFIRQGNMIKRDTTMIPLQ---QTEEEEFY-TFIGQFY--SLNQHILPKEVHVPRNLD------KEMIQSVVDTKIV--QPARGPKKDMVDL 342

               380                 400                 420                 440                 460
Hp_26695      AMKNAQEIFSQEKTSNEDLILEEARSLFKLECM--------P--YRVEIFDTSHHSSSQCVGGMVVYENNAFQKNSYRRYHLKG---SDEYTQ 438
Cj_NCTC_11168 AFQNALLNIEKEQKNHDFTIQKELKSYFELENL--------P--NDIEIFDNSHLQGVANVGAMVTYRINSWDKSKYRKFHLKH---KNDYDQ 437
Ec_K12        ARTNAATALTS-KLSQQSTVHQRLTALASVLKL--------PEVKRMECFDISHTMGEQTVASCVVFDANGPLRAEYRRYNITGITPGDDYAA 441
Sa_N315       AAHNAKVSLNN---KFELISRDESRTIKAIEELGTQMGIQTP--IRIEAFDNSNIQGVDPVSAMVTFVDGKPDKKNYRKYKIKTVKGPDDYKS 430

               480                 500                 520                 540
Hp_26695      MSELLTRR---ALDFAKEPPPNLWVIDGGRAQLNIALEILK-SSGSFVE-----VIAISKEKRDSKAYRSKGGAKDIIHTPSDT-FKLLPSDK 521
Cj_NCTC_11168 MREVLTRR---ALDFDKIPPPDLWLIDGGKALLDLAKEIIV-SSGVNVD-----ILAISKEKIDAKAHRAKGGAKDKIHSLKGE-FSLSINDK 520
Ec_K12        MNQVLRRRYGKAIDDSKI--PDVILIDGGKGQLAQAKNVFA-ELDVSWDKNHPLLLGVAKGA------DRKAGLETLFFEPEGEGFSLPPDSP 525
Sa_N315       MREVVRRRYSRVLN-EGLPLPDLIIVDGGKGHMNGVIDVLQNELGLDIP-----VAGLQKND--------KHQTSELLYGASAEIVPLKKNSQ 509

               560                 580                 600                 620                 640
Hp_26695      RLQWVQKLRDESHRYAINFHRSTKLKNMKQIALLKEKGIGEASVKKLLDYFGSFEAIEKASEQEKNAV------LKKRI------ 594
Cj_NCTC_11168 KLQFLQKLRDEAHRFAISFHQNTKKKQDLKSSKLANLGLSSGVMQKLLAYYGNFESIYKADFKDLTML--VGRKAAQKIKEN--- 600
Ec_K12        ALHVIQHIRDESHDHAIGGHRKKRAKVKNTSSLETIEGVGPKRRQMLLKYMGGLQGLRNASVEEIAKVPGISQGLAEKIFWSLKH 610
Sa_N315       AFYLLHRIQDEVHRFAITFHRQTRQKTGLKSILDDIDGIGNKRKTLLRSFGSIKKMKEATLEDFKNI-GIPENVAKNLHEQLHK 593
```

D

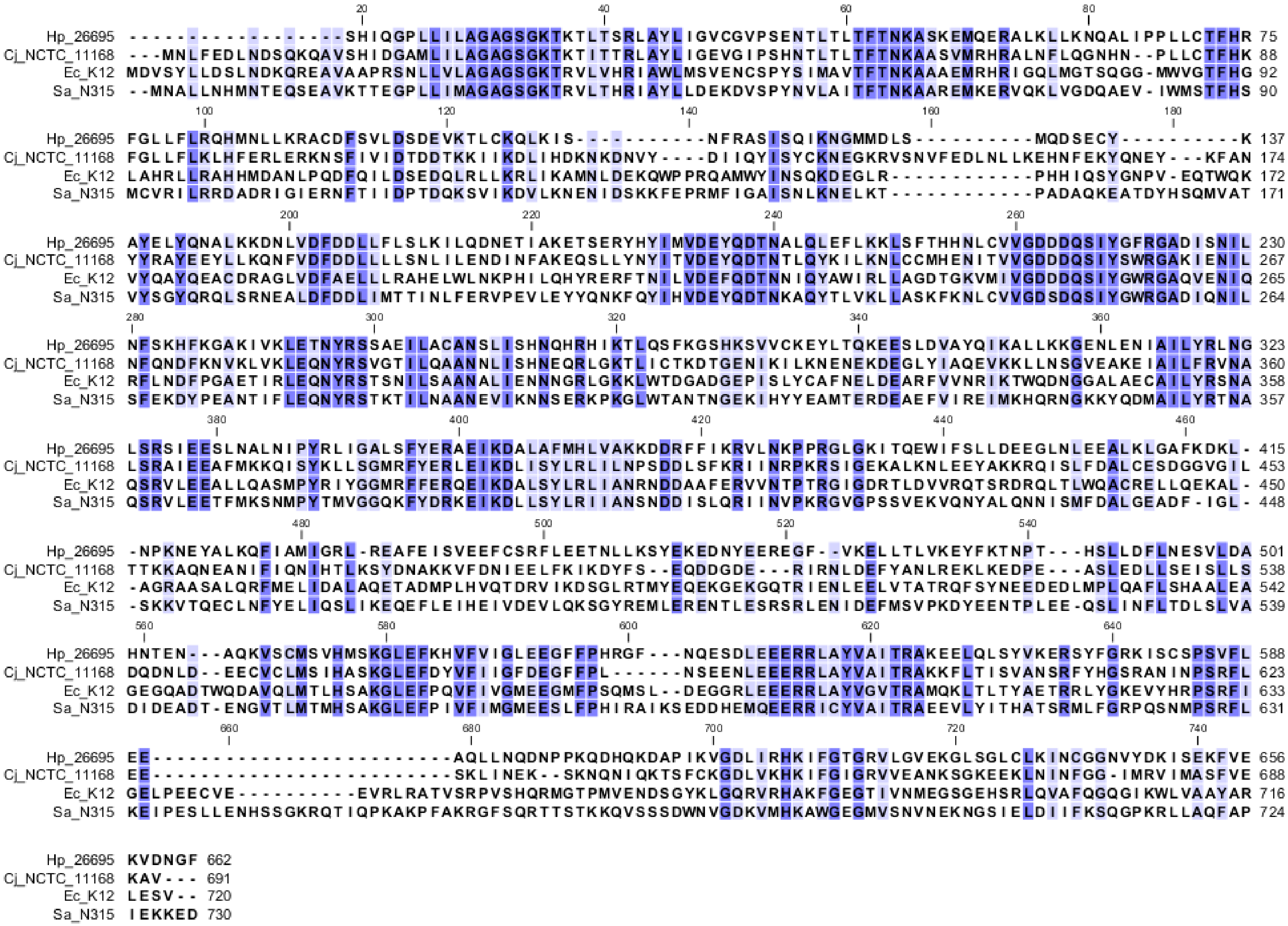
20 40 60 80
Hp_26695 ------------------SHIQGPLLILAGAGSGKTKTLTSRLAYLIGVCGVPSENTLTLTFTNKASKEMQERALKLLKNQALIPPLLCTFHR 75
Cj_NCTC_11168 ---MNLFEDLNDSQKQAVSHIDGAMLILAGAGSGKTKTITTRLAYLIGEVGIPSHNTLTLTFTNKAASVMRHRALNFLQGNHN--PLLCTFHK 88
Ec_K12 MDVSYLLDSLNDKQREAVAAPRSNLLVLAGAGSGKTRVLVHRIAWLMSVENCSPYSIMAVTFTNKAAAEMRHRIGQLMGTSQGG-MWVGTFHG 92
Sa_N315 --MNALLNHMNTEQSEAVKTTEGPLLIMAGAGSGKTRVLTHRIAYLLDEKDVSPYNVLAITFTNKAAREMKERVQKLVGDQAEV-IWMSTFHS 90
100 120 140 160 180
Hp_26695 FGLLFLRQHMNLLKRACDFSVLDSDEVKTLCKQLKIS-----------NFRASISQIKNGMMDLS----------MQDSECY----------K 137
Cj_NCTC_11168 FGLLFLKLHFERLERKNSFIVIDTDDTKKIIKDLIHDKNKDNVY----DIIQYISYCKNEGKRVSNVFEDLNLLKEHNFEKYQNEY---KFAN 174
Ec_K12 LAHRLLRAHHMDANLPQDFQILDSEDQLRLLKRLIKAMNLDEKQWPPRQAMWYINSQKDEGLR------------PHHIQSYGNPV-EQTWQK 172
Sa_N315 MCVRILRRDADRIGIERNFTIIDPTDQKSVIKDVLKNENIDSKKFEPRMFIGAISNLKNELKT------------PADAQKEATDYHSQMVAT 171
200 220 240 260
Hp_26695 AYELYQNALKKDNLVDFDDLLFLSLKILQDNETIAKETSERYHYIMVDEYQDTNALQLEFLKKLSFTHHNLCVVGDDDQSIYGFRGADISNIL 230
Cj_NCTC_11168 YYRAYEEYLLKQNFVDFDDLLLLSNLILENDINFAKEQSLLYNYITVDEYQDTNTLQYKILKNLCCMHENITVVGDDDQSIYSWRGAKIENIL 267
Ec_K12 VYQAYQEACDRAGLVDFAELLLRAHELWLNKPHILQHYRERFTNILVDEFQDTNNIQYAWIRLLAGDTGKVMIVGDDDQSIYGWRGAQVENIQ 265
Sa_N315 VYSGYQRQLSRNEALDFDDLIMTTINLFERVPEVLEYYQNKFQYIHVDEYQDTNKAQYTLVKLLASKFKNLCVVGDSDQSIYGWRGADIQNIL 264
280 300 320 340 360
Hp_26695 NFSKHFKGAKIVKLETNYRSSAEILACANSLISHNQHRHIKTLQSFKGSHKSVVCKEYLTQKEESLDVAYQIKALLKKGENLENIAILYRLNG 323
Cj_NCTC_11168 NFQNDFKNVKLVKLEQNYRSVGTILQAANNLISHNEQRLGKTLICTKDTGENIKILKNENEKDEGLYIAQEVKKLLNSGVEAKEIAILFRVNA 360
Ec_K12 RFLNDFPGAETIRLEQNYRSTSNILSAANALIENNNGRLGKKLWTDGADGEPISLYCAFNELDEARFVVNRIKTWQDNGGALAECAILYRSNA 358
Sa_N315 SFEKDYPEANTIFLEQNYRSTKTILNAANEVIKNNSERKPKGLWTANTNGEKIHYYEAMTERDEAEFVIREIMKHQRNGKKYQDMAILYRTNA 357
380 400 420 440 460
Hp_26695 LSRSIEESLNALNIPYRLIGALSFYERAEIKDALAFMHLVAKKDDRFFIKRVLNKPPRGLGKITQEWIFSLLDEEGLNLEEALKLGAFKDKL- 415
Cj_NCTC_11168 LSRAIEEAFMKKQISYKLLSGMRFYERLEIKDLISYLRLILNPSDDLSFKRIINRPKRSIGEKALKNLEEYAKKRQISLFDALCESDGGVGIL 453
Ec_K12 QSRVLEEALLQASMPYRIYGGMRFFERQEIKDALSYLRLIANRNDDAAFERVVNTPTRGIGDRTLDVVRQTSRDRQLTLWQACRELLQEKAL- 450
Sa_N315 QSRVLEETFMKSNMPYTMVGGQKFYDRKEIKDLLSYLRIIANSNDDISLQRIINVPKRGVGPSSVEKVQNYALQNNISMFDALGEADF-IGL- 448
480 500 520 540
Hp_26695 -NPKNEYALKQFIAMIGRL-REAFEISVEEFCSRFLEETNLLKSYEKEDNYEEREGF--VKELLTLVKEYFKTNPT---HSLLDFLNESVLDA 501
Cj_NCTC_11168 TTKKAQNEANIFIQNIHTLKSYDNAKKVFDNIEELFKIKDYFS--EQDDGDE---RIRNLDEFYANLREKLKEDPE---ASLEDLLSEISLLS 538
Ec_K12 -AGRAASALQRFMELIDALAQETADMPLHVQTDRVIKDSGLRTMYEQEKGEKGQTRIENLEELVTATRQFSYNEEDEDLMPLQAFLSHAALEA 542
Sa_N315 -SKKVTQECLNFYELIQSLIKEQEFLEIHEIVDEVLQKSGYREMLERENTLESRSRLENIDEFMSVPKDYEENTPLEE-QSLINFLTDLSLVA 539
560 580 600 620 640
Hp_26695 HNTEN---AQKVSCMSVHMSKGLEFKHVFVIGLEEGFFPHRGF---NQESDLEEERRLAYVAITRAKEELQLSYVKERSYFGRKISCSPSVFL 588
Cj_NCTC_11168 DQDNLD--EECVCLMSIHASKGLEFDYVFIIGFDEGFFPL------NSEENLEEERRLAYVAITRAKKFLTISVANSRFYHGSRANINPSRFL 623
Ec_K12 GEGQADTWQDAVQLMTLHSAKGLEFPQVFIVGMEEGMFPSQMSL--DEGGRLEEERRLAYVGVTRAMQKLTLTYAETRRLYGKEVYHRPSRFI 633
Sa_N315 DIDEADT-ENGVTLMTMHSAKGLEFPIVFIMGMEESLFPHIRAIKSEDDHEMQEERRICYVAITRAEEVLYITHATSRMLFGRPQSNMPSRFL 631
660 680 700 720 740
Hp_26695 EE-------------------------AQLLNQDNPPKQDHQKDAPIKVGDLIRHKIFGTGRVLGVEKGLSGLCLKINCGGNVYDKISEKFVE 656
Cj_NCTC_11168 EE-------------------------SKLINEK--SKNQNIQKTSFCKGDLVKHKIFGIGRVVEANKSGKEEKLNINFGG-IMRVIMASFVE 688
Ec_K12 GELPEECVE----------EVRLRATVSRPVSHQRMGTPMVENDSGYKLGQRVRHAKFGEGTIVNMEGSGEHSRLQVAFQGQGIKWLVAAYAR 716
Sa_N315 KEIPESLLENHSSGKRQTIQPKAKPFAKRGFSQRTTSTKKQVSSSDWNVGDKVMHKAWGEGMVSNVNEKNGSIELDIIFKSQGPKRLLAQFAP 724
Hp_26695 KVDNGF 662
Cj_NCTC_11168 KAV--- 691
Ec_K12 LESV-- 720
Sa_N315 IEKKED 730
