# Supplementary material for: The nucleotide excision repair (NER) system of Helicobacter pylori: Role in mutation prevention and chromosomal import patterns after natural transformation
Source: BMC Microbiol. 2012 May 6;12:67. doi: 10.1186/1471-2180-12-67 (PMC3438104; doi:10.1186/1471-2180-12-67)
Supplement: Additional file 4 — Table S1. Bacterial strains [12,21,39,40]. Table S2. Oligonucleotide primers and PCR products used in this study [12,44]. Table S3. Plasmids used in this study [12,23,43-45,52]. [file 1471-2180-12-67-S4.doc]

Moccia et al., Supporting Tables

Table S1: Bacterial strains.

| **Strain** | **Genotype** | **Source** |
| --- | --- | --- |
| ***Escherichia coli*** | | |
| DH5α | F^-^, φ80d*lacZ*ΔM15, Δ(*lacZYA-argF*)U169, *deoR*, *recA*1, *endA*1, *hsdR*17(rk^-^, mk^+^), *phoA*, *supE*44, λ^-^, *thi*-1, *gyrA*96, *relA*1 | [1] |
| MC1065 | *E. coli* K-12 *leu*B6 Δ(*lac*JPOZY)X74 *trpC*9830 *strA* | [2] |
| ***Helicobacter pylori*** | | |
| 26695 | *H. pylori* wild type strain | [3] |
| J99-R3 | Derivative of J99; A to T mutation at position 1618 in *rpoB*; Rif^r^ | [4] |
| 26695*uvrA* | HP0705 (excinuclease ABC subunit A) from Hp 26695 inactivated with *aphA-3*’-III | This study |
| 26695*uvrA*comp | 26695*uvrA* strain complemented by introduction of uvrA plus upstream sequences into *rdxA* locus, using suicide plasmid pSUS3009 | This study |
| 26695*uvrB* | HP1114 (excinuclease ABC subunit B) from Hp 26695 inactivated with *aphA-3*’-III | This study |
| 26695*uvrB*comp | 26695*uvrB* strain complemented by introduction of *uvrB* into *ureAB* locus, using suicide plasmid pSUS2646 | This study |
| 26695*uvrC* | HP0821 (excinuclease ABC subunit C) from Hp 26695 inactivated with *cat* | This study |
| 26695*uvrC*comp | 26695*uvrC* strain complemented by introduction of *uvrC* into *ureAB* locus, using suicide plasmid pSUS2628 | This study |
| 26695Δ*uvrD* | HP1478 (DNA helicase II) from Hp 26695 inactivated with *aphA-3*’-III | This study |
| 26695*recA* | HP0153 (recombinase) from Hp 26695 inactivated with *aphA-3*’-III | [4] |

Table S2: Oligonucleotide primers and PCR products used in this study.

| **Primer** | **Target gene** | **5’ → 3’ sequence** | **RS*** | **Application** | **Source** |
| --- | --- | --- | --- | --- | --- |
| HPuvrA-1s | HP0705 (*uvrA*) | ataggatccATTATTCAAGGGGCTAGGG | *Bam*HI | Cloning | This study |
| HPuvrA-2s |  | ataggatccTATCCACAACAAAATCCGC | *Bam*HI |  |  |
| HPuvrA-3s |  | ataagatctTGAAGGAAAAACTCAAAGGC | *Bgl*II | Inverse PCR |  |
| HPuvrA-4s |  | ataagatctATTTTAGATCCTAACACGCC | *Bgl*II |  |  |
| HPuvrA_BamHI | HP0705 (*uvrA*) | agaggatccTTGGTGCCTCCTGGATTATG | *BamHI* | Functional complementation | This study |
| HPuvrA_BglII |  | ataagatctCTATTTCAATTCCAAAGCTA | *BglII* |  | This study |
| HPuvrB-1PstI | HP1114 (*uvrB*) | gcgctgcagCAAGCCATAGAAGCCTTGAC | *Pst*I | Cloning | This study |
| HPuvrB-2PstI |  | gcgctgcagTCTCTCAAACGCATCGCTTC | *Pst*I |  |  |
| HPuvrB-3BglII |  | ataagatctGTTTCGTTAGGGGCTTTACC | *Bgl*II | Inverse PCR |  |
| HPuvrB-4BglII |  | ataagatctGAGCAAATCATTCGCCCTAC | *Bgl*II |  |  |
| uvrBcomp-1s | HP1114 (*uvrB*) | atatctagaATGCCCTTATTTGATTTAA | *Xba*I | Functional complementation | This study |
| uvrBcomp-3s |  | atatctagaTTAAAGCGTTCTTAATTGA | *Xba*I |  |  |
| HPuvrC-1PstI | HP0821 (*uvrC*) | gcgctgcagTAACAGCAGTGGCGTGTATC | *Pst*I | Cloning | This study |
| HPuvrC-2PstI |  | gcgctgcagACGCTGGCTTCTCCTATACC | *Pst*I |  |  |
| HPuvrC-3BglII |  | ataagatctCTGGCATCAAAGGCAAATGC | *Bgl*II | Inverse PCR |  |
| HPuvrC-5 |  | GAACGCTTGCTCTAATGAAAC |  |  |  |
| HPuvrCcomp-1s | HP0821 (*uvrC*) | atatctagaTGGCTGATTTATTGTCCAGTT | *Xba*I | Functional complementation | This study |
| HPuvrCcomp-2s |  | atacccgggCACAAGCCCTATATTCAATC | *Sma*I |  |  |
| uvrD-1s_CM | HP1478 (Δ*uvrD*) | gcgctgcagATTACGATGCGAGTATCCGC | *Pst*I | Cloning | This study |
| uvrD-2s_CM |  | gcgctgcagGAGCTGCTCAACACTTTTGC | *Pst*I |  |  |
| uvrD-5s_CM |  | ataagatctGGCTCGTTAAAGTCTTAGTC | *Bgl*II | Inverse PCR |  |
| uvrD-6s_CM |  | ataagatctCTCTTATGTGAAAGAGCGTTC | *Bgl*II |  |  |
| HPrecA-1s | HP0153 (*recA*) | ataggatccGGCAATAGATGAAGACAAAC | BamHI | Cloning | [4] |
| HPrecA-2s |  | aatggatccACTCCATTTCTTCTAAAGGC | BamHI |  |  |
| HPrecA-3s |  | ataagatctCGTCATGCCAATCTTCATTC | BglII | Inverse PCR |  |
| HPrecA-4s |  | ttaagatctGGAGTCCAGAGACTACAAC | BglII |  |  |
| pcat-1 | *cat* cassette | AACAGCTATGACCATGATTACG | __ | Cloning | [5] |
| pCAT-12NdeI |  | agacatatgGATATCGCATGCCTGCAGAG | *Nde*I |  |  |
| pcat-2 |  | agaggatccGATATCGCATGCCTGCAGAG | *Bam*HI |  |  |
| HPrpoB-1 | HP1198 (*rpoB*) | CCCAACAGATTTAGAAGT | __ | *rpoB* sequencing | [4] |
| HPrpoB-3 |  | ATGTGCCTGATTACATCACGAC |  |  |  |
| HPrpoB-4 |  | TTGGCGCTGCATGTTAGTCC |  |  |  |
| HPrpoB-5 |  | GGTAGCCGCATCGCTCATTC |  |  |  |
| HPrpoB-6 |  | TTCCCTAACGCTAACTCGC |  |  |  |
| HPrpoB-9w |  | AGACGCYAATCARAGAATGG |  |  |  |
| HPrpoB-10 |  | CATCAATCTTGCCCTGATTG |  |  |  |
| HPrpoB-IscrX | *rpoB* | CCTTTGAGTGAAGTTCCGTA | __ | Import screening | [4] |

* RS: Restriction sites.

Table S3: Plasmids used in this study.

| **Plasmids** | **Genotype** | **Source** |
| --- | --- | --- |
| pADC | Amp^r^, Cm^r^ *H. pylori* *ureAB* fragment in pUC19 with *cat* cassette | [6] |
| pBHpC8 | Source of the *cat* cassette | [5] |
| pILL 600 | Source of the *aphA-3´-III* cassette | [7] |
| pUC18 | Amp^r^, Colx101, MCS within *lacZ*: blue/white selection | [8] |
| pUvrDKm | Amp^r^, pGEMT-Easy derivative containing *uvrD*::*aphA* | [9] |
| pSUS1700 | Amp^r^, pUC18 derivative containing *uvrA* | This study |
| pSUS1703 | Amp^r^, Km^r^, pSUS1700 derivative with a *uvrA::aphA-3´-III* disruption | This study |
| pSUS2503 | Amp^r^, pUC18 derivative containing *recA* | [4] |
| pSUS2511 | Amp^r^, Km^r^, pSUS2503 derivative with a *recA::aphA3´-III* disruption | [4] |
| pSUS2625 | Amp^r^, pUC18 derivative containing *uvrB* | This study |
| pSUS2627 | Amp^r^, Km^r^, pSUS2625 derivative with a *uvrB::aphA-3´-III* disruption | This study |
| pSUS2628 | Amp^r^, pUC18 derivative containing *uvrC* | This study |
| pSUS2630 | Amp^r^, Cm^r^, pSUS2628 derivative with a *uvrC::cat* disruption | This study |
| pSUS2632 | Amp^r^, Km^r^, *H. pylori ureAB* fragment in pUC19 with *aphA-3´-III* cassette | This study |
| pSUS2640 | Amp^r^, pUC18 derivative containing *uvrD* | This study |
| pSUS2642 | Amp^r^, Km^r^, pSUS2640 derivative with a *uvrD::aphA-3´-III* disruption (full deletion) | This study |
| pSUS2644 | Amp^r^, Km^r^, pSUS2632 derivative with *uvrC* | This study |
| pSUS2646 | Amp^r^, Cm^r^, pADC derivative with *uvrB* | This study |
| pSUS3009 | Amp^r^, Cm^r^, pCJ535 derivative with *uvrA* | This study |

Reference List

1. Hanahan D: **Studies on transformation of *Escherichia coli* with plasmids.** *J Mol Biol* 1983, **166:**557-580.

2. Casadaban MJ, Cohen SN: **Analysis of gene control signals by DNA fusion and cloning in *Escherichia coli*.** *J Mol Biol* 1980, **138:**179-207.

3. Tomb JF, White O, Kerlavage AR, Clayton RA, Sutton GG, Fleischmann RD, Ketchum KA, Klenk HP, Gill S, Dougherty BA et al.: **The complete genome sequence of the gastric pathogen *Helicobacter pylori*.** *Nature* 1997, **388:**539-547.

4. Kulick S, Moccia C, Didelot X, Falush D, Kraft C, Suerbaum S: **Mosaic DNA imports with interspersions of recipient sequence after natural transformation of *Helicobacter pylori*.** *PLoS One* 2008, **3:**e3797.

5. Ge Z, Hiratsuka K, Taylor DE: **Nucleotide sequence and mutational analysis indicate that two *Helicobacter pylori* genes encode a P-type ATPase and a cation-binding protein associated with copper transport.** *Mol Microbiol* 1995, **15:**97-106.

6. Huang S, Kang J, Blaser MJ: **Antimutator role of the DNA glycosylase *mutY* gene in *Helicobacter pylori*.** *J Bacteriol* 2006, **188:**6224-6234.

7. Labigne-Roussel A, Courcoux P, Tompkins L:  **Gene disruption and replacement as a feasible approach for mutagenesis of *Campylobacter jejuni*.** *J Bacteriol* 1988, **170:**1704-1708.

8. Yanisch-Perron C, Vieira J, Messing J: **Improved M13 phage cloning vectors and host strains: nucleotide sequences of the M13mp18 and pUC19 vectors.** *Gene* 1985, **33:**103-119.

9. Kang J, Blaser MJ: **UvrD helicase suppresses recombination and DNA damage-induced deletions.** *J Bacteriol* 2006, **188:**5450-5459.
